# Modeling the Aneuploidy Control of Cancer

**DOI:** 10.1186/1471-2407-10-346

**Published:** 2010-07-01

**Authors:** Yao Li, Arthur Berg, Louie R Wu, Zhong Wang, Gang Chen, Rongling Wu

**Affiliations:** 1 Department of Statistics, West Virginia University, Morgantown, WV 26506, USA; 2 Center for Statistical Genetics, Pennsylvania State University, Hershey, PA 17033, USA; 3 Penn State Hershey Cancer Institute, Pennsylvania State College of Medicine, Hershey, PA 17033, USA; 4 California Institute of Technology, Pasadena, CA 91125, USA

## Abstract

**Background:**

Aneuploidy has long been recognized to be associated with cancer. A growing body of evidence suggests that tumorigenesis, the formation of new tumors, can be attributed to some extent to errors occurring at the mitotic checkpoint, a major cell cycle control mechanism that acts to prevent chromosome missegregation. However, so far no statistical model has been available quantify the role aneuploidy plays in determining cancer.

**Methods:**

We develop a statistical model for testing the association between aneuploidy loci and cancer risk in a genome-wide association study. The model incorporates quantitative genetic principles into a mixture-model framework in which various genetic effects, including additive, dominant, imprinting, and their interactions, are estimated by implementing the EM algorithm.

**Results:**

Under the new model, a series of hypotheses tests are formulated to explain the pattern of the genetic control of cancer through aneuploid loci. Simulation studies were performed to investigate the statistical behavior of the model.

**Conclusions:**

The model will provide a tool for estimating the effects of genetic loci on aneuploidy abnormality in genome-wide studies of cancer cells.

## Background

In recent years, there has been a wealth of literature on the development of statistical methods for genetic analysis of complex diseases, such as cancer [1,2]. These methods, mostly founded on rigorous statistical theory and models, have been instrumental in the analysis and modeling of genetic data, leading to the identification of significant genetic variants involved in pathogenesis [3,4]. However, many existing statistical methods neglect biological principles refreshed and updated from the latest scientific discoveries obtained by using new genomic technologies. A lack of the integration between statistics and biology will significantly limit our detection and characterization of the new genetic underpinnings of a disease. The motivation of this study is to develop a novel statistical model for detecting the genetic control of cancer through chromosomal loci predisposing to aneuploidy.

Aneuploidy occurs when an individual has an abnormal number of chromosomes. Partial or whole chromosomes may be duplicated or missing in individuals with this condition. Cytological studies show that aneuploidy is one of the most pronounced differences between normal and cancer cells [5]. However, debates have arisen over how aneuploid cells are produced and whether or not they are a cause or consequence of tumorigenesis [6,7]. A growing body of evidence from molecular genetic studies supports a role of aneuploidy in the genetic underpinning of cancer [6,8-12]. According to extensive work by Duesberg and his group, the impact of aneuploidy on cancer is embodied in the following aspects:

(1) Aneuploidy is confirmed to generate abnormal phenotypes, such as Down syndrome in humans and cancer in animals;

(2) The degree of aneuploidy is correlated with phenotype abnormality;

(3) Since aneuploidy imbalances the highly balance-sensitive components of the spindle apparatus, it destabilizes symmetrical chromosome segregation;

(4) Both non-genotoxic and genotoxic carcinogens can cause aneuploidy by physical or chemical interaction with mitosis proteins.

Similar to point (2), there is additional evidence that cancer-specific phenotypes result when aneuploidy exceeds a certain threshold [13,14]. Kops et al. [15] outlined the cytological mechanisms for aneuploid formation from checkpoint signalling. Normally, chromosome mis-segregation can be prevented at the mitotic checkpoint by delaying cell-cycle progression through mitosis until all chromosomes have successfully made spindle-microtubule attachments, but a defect in the mitotic checkpoint can generate aneuploidy, facilitate tumorigenesis, and can cause increased resistance to anti-cancer therapies [16].

The statistical model developed to detect cancer genes is constructed with a random sample of aneuploid patients with cancer drawn from a natural population. At an aneuploid locus, polyploids occur because of the duplication of one or two parental chromosomes and, thus, the model can be formulated to test the genetic imprinting of alleles due to their different parental origins.

If the aneuploidy hypothesis is continuously confirmed, this model will provide a timely tool to quantify the genetic effects of aneuploidy loci on cancer susceptibility by integrating the genetic data from the cancer genome project. Also, by comparing with the model for detecting somatic mutations, this new model will help to determine the relative importance of the aneuploidy and mutation hypotheses in cancer studies.

## Methods

### Study Design

Suppose there is a normal human diploid population which is at Hardy-Winerberg equilibrium (HWE). Some individuals in this population form cancer owing to particular regions of their chromosomes multiplied to form a triploid, tetraploid, or a polyploid of any higher order. To simply describe our model, we only consider a triploid. As proven below, the population after chromosomal multiplication will deviate from HWE. We assume that a total of *n *cancer patients are randomly sampled from this population. Each sampled patient is a triploid at a particular aneuploid locus. We genotype all these patients at duplicated chromosomal segments with molecular markers, although the parental origin of chromosomal duplication is unknown. A phenotype that defines cancer is measured for all subjects. A model will be derived to distinguish between the genetic effects of alleles inherited from the maternal (M) and paternal parents (P).

### Chromosome Duplication

#### Triploid Model

Consider a gene of interest **A**, with two alleles *A *and *a*, on a chromosome (say chromosome 3). Figure describes the process of a pair of normal chromosomes that are duplicated into a triploid for a portion of chromosome 3. For a normal diploid, the genotypes at this gene may be *AA*, *Aa*, or *aa*. Considering parent-specific origins of alleles, we use *A*|*a*, *A*|*a*, *a*|*A*, and *a*|*a *to denote the configurations of these genotypes, respectively, where the left- and right-side alleles of the vertical lines represent two alleles from different parents. Of these, configurations *A*|*a *and *a*|*A *are genotypically observed as the same genotype *Aa*. When the chromosomal segment that harbors this gene are duplicated for only one single chromosome, triploids with two copies from one parent and the third copy from the other parent will result. It is possible that a single chromosome derived from maternal and paternal parents may both be duplicated, but with a different frequency. Thus, through such a duplication, four configurations in the normal diploid will form a total of eight triploid configurations, which are classified into four different genotypes:

(1) *AAA *including configurations *AA*|*A*, duplicated from the left-side parent, and *A*|*AA*, duplicated from the rightside parent, of configuration *AA*;

(2) *AAa *including configurations *AA*|*a *duplicated from the left-side parent of configuration *A*|*a *and *a*|*AA *duplicated from the right-side parent of configuration *a*|*A*;

(3) *Aaa *including configurations *aa*|*A *duplicated from the left-side parent of configuration *a*|*A *and *A*|*aa *duplicated from the right-side parent of configuration *A*|*a*;

(4) *aaa *including configurations *aa*|*a*, duplicated from the left-side parent, and *a*|*aa*, duplicated from the right-side parent, of configuration *aa*.

Let *p *and *q *(*p *+ *q *= 1) are the allele frequencies of *A *and *a *in the original population before chromosome duplication. For a natural population at HWE, genotype frequencies can be expressed as *p*^2 ^for genotype *AA*, 2*pq *for genotype *Aa*, and *q*^2 ^for genotype *aa*.

#### Theorem

*For an HWE diploid population, chromosome duplication operating on particular loci can violate the equilibrium status of the population*.

*Proof*: Let *g *and *h *denote a proportion of allele *A *and *a *that is duplicated, respectively. Thus, of diploid genotype *AA*, a proportion *g *will become *AAA*, with the remaining proportion 1 - *g *unduplicated. Similarly, a proportion *h *and 1 - *h *will be aaa and aa after duplication for diploid genotype *aa*. Diploid genotype *Aa *will have three possibilities, *AAa *with a proportion of *g*, *Aaa *with a proportion of *h*, and *Aa *with a proportion of 1 - *g *- *h*. In a duplicated population purely composed of triploids, we will have allele frequencies for *A *and *a*, respectively, as

The genotype frequency of triploid *AAA *in the duplicated population is expressed as

Similarly, we have the genotype frequency of triploid *aaa *as

Thus, unless  and , the duplicated population will be at Hardy-Weinberg disequilibrium.

This theorem shows that traditional HWE theory for population genetic studies will not be useful for cancer gene identification. Meanwhile, this theorem provides a foundation for deriving a statistical model to conduct genome-wide association studies of cancer.

### Quantitative Genetic Parameters

The cancer patients sampled are purely composed of triploids at an aneuploid locus. Each patient is typed for DNA-based markers at aneuploid loci and also phenotyped for a cancer trait. Let *n_k _*denote the observed number of triploid genotype *k *(*k *= 1 for *AAA*, 2 for *AAa*, 3 for *Aaa*, and 4 for *aaa*). The total number of patients sampled is . It has proven that chromosomal duplication may violate the Hardy-Weinberg equilibrium of the population. Thus, genotype frequencies, *P_k_*, are expressed as the products of allele frequencies plus disequilibrium parameters. Let *D*_1 _and *D*_2 _denote the Hardy-Weinberg disequilibrium coefficients associated with allele *A *and *a*, respectively, at the duplicated gene. Thus, the frequencies of four genotypes can be expressed in terms of allele frequencies and disequilibria (Table 1).

**Table 1:** The changes of genotypes and genotype frequencies after chromosomal duplication.

Non-duplicated			Duplicated		
		
Genotype	Frequency	Duplication	Genotype	Frequency	Observation
*AA*	*p*^2^	⇒	*AAA*		*n*_1_
*Aa*	2*pq*	⇒	*AAa*		*n*_2_
			*Aaa*		*n*_3_
*aa*	*q*^2^	⇒	*aaa*		*n*_4_

The same triploid genotype at the duplicated gene may have different values when the expression of its alleles depends on the origin of parents. For example, triploid genotype *AAA *may be formed from normal diploid genotype *AA *when either the maternally-(M) or paternally-derived allele (P) is doubled. Thus, the configuration of genotype *AAA *can be either *A_M_A_M_A_F _*or *A_M_A_F_A_F_*, where the subscripts denote the parental origin of alleles. Table 2 gives the genotypic values (*μ*_*k*1_, *μ*_*k*2_) of two possible configurations of each triploid genotype. These genotypic values are partitioned into eight different components, the overall mean (*μ*), additive dominant genetic effect (*a*), dominance genetic effects of *AA *over *a *(*d*) and *A *over *aa *(*d'*), genetic imprinting effects due to different origins of alleles (*λ*), and interactions between the additive and imprinting effects (*I*_*aλ*_), the *d *dominance and imprinting effects (*I*_*dλ*_), and the *d' *dominance and imprinting effects (*I*_*d'λ*_), respectively.

**Table 2:** Genotypic values and proportions of different configurations of a triploid genotype at a duplicated gene.

Duplicated Genotype	Configuration	Genetic Value	Duplication Rate
*AAA*			
*AAa*			
*Aaa*			
*aaa*			

For each triploid genotype, the relative proportions of two underlying configurations can be different, depending on the rate of the duplication of parent-specific chromosomes. Let *u *and 1 - *u *be the proportions of the duplication of allele *A *derived from the maternal and paternal parents, respectively. Similarly, let *v *and 1 - *v *be the proportions of the duplication of allele *a *derived from the maternal and paternal parents, respectively (Table 2). These proportions can be estimated from genotype data.

### Estimation

It is straightforward to estimate the frequency of a triploid genotype with genotype observations using(1)

which is derived from a polynomial likelihood. The EM algorithm is implemented to estimate the allele frequencies ( and ) and HWD coefficients from the triploid genotype observations of the aneuploid population sampled (Table 1). It is described as follows:

In the E step, we calculate the proportion of an allele within a triploid genotype using(2)

for allele *A*, and(3)

for allele *a*.

In the M step, the allele frequencies are then estimated with the following equations:(4)(5)

and the HWD coefficients are calculated by(6)(7)

To estimate the duplication rate and genotypic values for each configuration, we need to formulate a mixture model because each triploid genotype contains two unknown configurations. The likelihood of genotype observations at the duplicated gene (Table 1) and phenotypic values (*y*) measured for all subjects is constructed as(8)

where  is the vector of unknown parameters, and  exp  (*k *= 1, ..., 4; *j *= 1, 2) is the normal distribution of the phenotypic trait with mean *μ_kj _*and variance *σ*^2^.

To obtain the maximum likelihood estimates (MLEs) of the parameters, we implement the EM algorithm to the likelihood (8). In the E step, the posterior probability with which a subject *i *with a specific triploid genotype has a configuration *j *is calculated using(9)

In the M step, by solving the loglikelihood equations, the parameters are estimated with the calculated posterior probabilities, i.e.,(10)(11)(12)(13)

A loop of the E and M steps is iterated between equations (9) and (10), (11), (12) and (13). Thus, the parameter estimates are obtained when the estimates converge to stable values. The MLEs of genetic effects can be obtained by solving a system of linear equations given in Table 2, i.e.,(14)(15)(16)(17)(18)(19)(20)

### Hypothesis Tests

How a duplicated gene deviates from Hardy-Weinberg equilibrium can be tested by formulating the null hypothesis as follows:

under which genotype frequencies can be estimated from the estimated allele frequencies using equation (1). The log-likelihood ratio calculated under the null and alternative hypotheses follows a χ^2^-distribution with 2 degrees of freedom. It is interesting to test the two disequilibria separately. Under the null hypothesis *H*_0 _: *D*_1 _= 0. genotype frequencies are estimated using equation (1), but with a constraint *P*_3 _= *p*^3^, in addition to constraint *P*_1_+*P*_2_+*P*_3_+*P*_4 _= 1. Similarly, genotype frequencies are estimated with a constraint *P*_4 _= *q*^3 ^for testing whether *D*_2 _= 0.

Whether the duplicated gene is significantly associated with cancer susceptibility can be tested using the null hypothesis *μ*_*kj *_≡ *μ *for *k *= 1, ..., 4; *j *= 1, 2. The additive effect and two types of dominance effects can be tested jointly or separately by formulating the relevant null hypotheses based on equations (14), (15), and (16). The imprinting effect and its interactions with additive and dominance effects can be tested by using the null hypothesis *H*_0 _: *λ *= 0, *H*_0 _: *I*_*aλ *_= 0, *H*_0 _: *I*_*dλ *_= 0, and *H*_0 _: *I*_*d'λ*_= 0 constructed with equations (**??**), (18), (18), and (19), respectively.

The model can also be used to test the significance of duplication rate for a parentspecific chromosome by formulating the null hypothesis *H*_0 _: *u *= 1 or *H*_0 _: *v *= 1. This information helps to understand the genetic structure and evolutionary process of cancer risk.

## Results

Simulation studies were used to investigate the statistical properties of the model in terms of estimation precision, power and false positive rates. We simulate a cancer population of triploids for a portion of chromosome. The allele frequencies at a triploid locus are  = 0.6 and  = 0.4. The HWD coefficients at this locus are assumed as *D*_1 _= 0:08, *D*_2 _= 0:06. By assuming the duplication rates of 0.3 and 0.4 for two parental chromosomes, respectively, the distribution of four different triploid genotypes *AAA*, *AAa*, *Aaa*, and *aaa *can be simulated. The phenotypic values of cancer traits were simulated by summing the additive, dominance, imprinting, and their interaction effects given with particular values and the errors of measurement within each triploid genotype following a normal distribution with variance scaled by a heritability of 0.05, 0.10, and 0.20, respectively. Different sample sizes, 400, 800, and 2,000 are considered.

The model was used to estimate allele frequencies, HWD, parent-specific duplication rates, and genetic effects for a cancer population (Table 3). As shown by small sampling errors of the estimates, allele frequencies can be precisely estimated with a modest sample size (400). A larger sample size (say 800) is needed to provide precise estimation of two HWD coefficients *D*_1 _and *D*_2_. Because duplication rates determine the mixture proportions for each triploid genotype, their estimates will be affected by the heritability level. If the cancer trait has a larger heritability, then a sample size of 400 will provide good estimates of duplication rates. For a less heritable trait, a large sample size (2000 or even more) is needed for good estimation. The additive effect can be generally well estimated, but the estimates of the dominant effects need much larger sample size. The estimation precision of the imprinting effect seems to be intermediate between that of the additive and dominant effects. It is interesting to see that the additive *× *imprinting interaction effect can be better estimated than the imprinting effect alone. It is hard to estimate the interactions between the dominant and imprinting effects unless an extremely large sample size (> 2000) is used. Overall, the impact of heritability is large than that of sample size, suggesting that it is important to allocate limited resource to measure phenotypes precisely rather than increase sample size simply.

**Table 3:** The estimates of population genetic parameters (*p*, *u*, *v*) and quantitative genetic parameters (*a*, *d*, *d'*, *λ*, *I*_*aλ*_, *I*_*dλ*_, *I*_*d'λ*_) from simulated data with different sample size and heritability combinations.

Sample Size	*H*^2^	*p*	*u*	*v*	*a*	*d*	*d^'^*	*λ*	*I*_*aλ*_	*I*_*dλ*_	*I*_*d'λ*_
True Value		0:6	0:3	0:4	0:8	0:5	0:4	0:5	0:4	0:5	0:3
400	0.05	0:5973	0:2974	0:3389	0:9378	0:7793	0:5928	0:7974	0:6869	0:9731	0:5860
		(0:0017)	(0:0149)	(0:0197)	(0:0468)	(0:1618)	(0:1052)	(0:0832)	(0:0590)	(0:2354)	(0:1218)
	0.1	0:6014	0:2803	0:3783	0:9252	0:5270	0:3571	0:6732	0:5647	0:6423	0:5066
		(0:0016)	(0:0183)	(0:0190)	(0:0404)	(0:1548)	(0:0662)	(0:0664)	(0:0447)	(0:1854)	(0:0750)
	0.2	0:6016	0:2873	0:4139	0:8333	0:5436	0:3354	0:5116	0:4308	0:4066	0:3433
		(0:0016)	(0:0161)	(0:0198)	(0:0274)	(0:0798)	(0:0554)	(0:0435)	(0:0294)	(0:1093)	(0:0481)
800	0.05	0:5980	0:2463	0:3449	1:0740	0:6989	0:3363	0:8930	0:7510	0:6963	0:5266
		(0:0012)	(0:0154)	(0:0227)	(0:0576)	(0:2247)	(0:1128)	(0:0909)	(0:0626)	(0:2694)	(0:1153)
	0.1	0:5993	0:2722	0:3783	0:8945	0:6552	0:4731	0:6004	0:5015	0:6375	0:5637
		(0:0013)	(0:0156)	(0:0210)	(0:0349)	(0:1199)	(0:0854)	(0:0606)	(0:0399)	(0:1565)	(0:0781)
	0.2	0:5988	0:2809	0:3763	0:8756	0:4041	0:4281	0:5925	0:4799	0:3461	0:3254
		(0:0011)	(0:0166)	(0:0194)	(0:0275)	(0:0781)	(0:0444)	(0:0379)	(0:0253)	(0:1035)	(0:0466)
2000	0.05	0:6011	0:2428	0:3656	1:0367	0:7602	0:3605	0:7556	0:6271	0:8159	0:4858
		(0:0007)	(0:0165)	(0:0209)	(0:0622)	(0:2001)	(0:0852)	(0:0995)	(0:0647)	(0:2416)	(0:1019)
	0.1	0:6008	0:2812	0:3805	0:8417	0:6676	0:4135	0:4838	0:4217	0:6970	0:5141
		(0:0007)	(0:0161)	(0:0211)	(0:0320)	(0:1337)	(0:0546)	(0:0537)	(0:0370)	(0:1585)	(0:0622)
	0.2	0:6001	0:2860	0:3771	0:8103	0:5607	0:4490	0:4614	0:3758	0:6301	0:3942
		(0:0008)	(0:0132)	(0:0185)	(0:0153)	(0:0511)	(0:0383)	(0:0297)	(0:0195)	(0:0699)	(0:0331)

The numbers in parentheses are the sampling errors of the estimates.

The power to detect the overall genetic effect and imprinting effect was investigated. In general, the model has great power for the identification of aneuploid loci causing cancer. To achieve adequate power for imprinting effect detection, a large sample size and/or large heritability is required. Overall, a sample size of 400 with a heritability of 0.2 can reach power of over 0.75 for the detection of imprinting effects. We also performed simulation studies to examine the false positive rates for detecting overall genetic effects and imprinting effects at aneuploid loci. It appears that in each case the false positive rates can be controlled to be below 5-10%.

## Discussion

Over the past 100 years since Theodor Boveri hypothesized that mitotic defects that result in tetraploidy promote oncogenesis [17], a tremendous concern has been given to explore the genetic cause of tumorigenesis. It has been partly established that aneuploidy has an effect on proliferation and survival of tumors [5]. The recent discovery of components of the mitotic checkpoint, as well as the realization that many of the classic tumour suppressors and oncogene products regulate mitotic progression, has renewed interest in the role of aneuploidy in tumorigenesis [10,15,16]. With the completion of the human genome projects and HapMap project, there is a pressing need for the development of statistical models for estimating the genetic effect of aneuploid loci on cancer risk.

In this article, we present a statistical strategy for detecting the genetic control of cancer traits through genotyping aneuploids of cancer cells. The model proposed presents two novelties. First, it has for the first time integrated the latest discovery of cancer genetic studies with statistical principles and directly pushed the modeling effort of cancer gene identification at the frontier of cancer biology. The experimental design used is founded on biologically relevant hypotheses from which data can be collected in an effective way. The derived closed forms for the EM algorithm to estimate various parameters will provide an efficient computation for any data set. Second, the model capitalizes on traditional quantitative genetic theory, allowing the partition of overall genetic control into different components. Particularly, we are able to estimate and test the effect of genetic imprinting on cancer risk [18,19] and, thus, draw a detailed picture of genetic control triggered from different parental chromosomes. The model can also characterize the interactions of additive and dominant effects with imprinting effects, helping to gain a better insight into the complexity of the genetic architecture of cancer.

We performed computer simulation to examine the statistical properties of the model. Results from simulation studies were investigated, from which an appropriate sample size is determined for a cancer trait with a particular heritability. Analyses of model power and false positive rates validated the possible usefulness of the model when practical data sets are available. Through a simple mathematical proof, we found that the Hardy-Weinberg equilibrium of an original population can be destroyed when some chromosomes are duplicated.

The idea of the model can be extended to several more complicated situations. First, the aneuploidy control of cancer may be derived from high-order aneuploid, such as tetraploids. A high-order polyploid not only contain more allelic combinations, but also a more amount of missing data due to the duplication of different chromosomes with unknown parental origins. To model the tetraploidy control of cancer, a more sophisticated algorithm is required to obtain efficient estimates of parameters. Second, different aneuploid loci responsible for cancer traits may be associated in the duplication population and interact in a coordinated manner. Modeling of multi-locus associations and multi-locus epistasis will deserve a further investigation although these pieces of information can better explain the genetic variation of cancer than single loci. Third, other factors, such as sex, race, and life style, also contribute to cancer. It is crucial to incorporate these factors and study the effects of each of them and their interactions with genes in tumorigenesis.

## Conclusion

We have derived a new statistical model for identifying genetic loci that control quantitative phenotypes of aneuploidy cancer through a genome-wide association study. We integrate quantitative genetic principles into the model, allowing the estimation of different types of genetic effects. The new model can generate a series of hypotheses tests about the explanation of the genetic control mechanisms of cancer through aneuploid loci. Although our model was explored merely from a theoretical perspective, specific experiments should be readily launched to collect the data according to the genetic design suggested. By analyzing such data, the new model should be able to uncover unique results, facilitating our understanding of how aneuploid processes are linked with cancer through genetic mediations.

## Competing interests

The authors declare that they have no competing interests.

## Authors' contributions

YL derived the equations and performed simulation studies. AB led and performed simulation studies. LW participated in simulation studies. ZW participated in simulation studies. GC interpreted the biological relevance of the model. RW conceived of the model and coordinated its design and simulation test. All authors read and approved the final manuscript.

**Figure 1:**
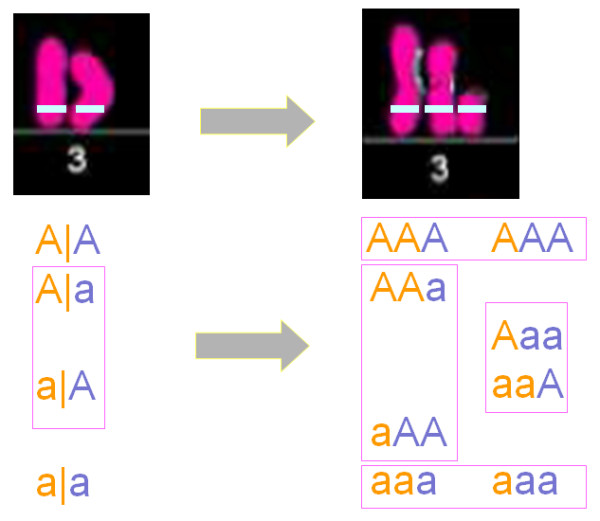
**Diagram for chromosome duplication and the resulting changes of genotypes at an aneuploid gene A**. Alleles in different colors denote their parent-specific origins separated by the vertical lines.

## Acknowledgements

We thank Dr. Justo Lorenzo Bermejo, Dr. George Heinze, Dr. Marek Kimmel, and Dr. Elizabeth Petty for their constructive comments which help to improve the manuscript. This work is supported by joint grant DMS/NIGMS-0540745 and a Penn State Cancer Institute Seed Grant.

## Pre-publication history

The pre-publication history for this paper can be accessed here:

http://www.biomedcentral.com/1471-2407/10/346/prepub
